# Renalase rs2296545 variant improve hypertension susceptibility by modifying binding affinity to catecholamines in obstructive sleep apnea

**DOI:** 10.1038/s41440-024-01850-0

**Published:** 2024-09-04

**Authors:** Hangdong Shen, Jundong Yang, Wenjun Xue, Zhicheng Wei, Lilin Li, Jian Guan, Xinyi Li, Xiaolin Wu

**Affiliations:** 1 Department of Otorhinolaryngology Head and Neck Surgery, Shanghai Sixth People’s Hospital Affiliated to Shanghai Jiao Tong University School of Medicine, Shanghai, China; 2 Shanghai Key Laboratory of Sleep Disordered Breathing, Shanghai, China; 3 Otorhinolaryngology Institute of Shanghai Jiao Tong University, Shanghai, China; 4 Department of Clinical Laboratory, Shanghai Children’s Hospital, Shanghai Jiaotong University, Shanghai, China; 5 Faculty of Medical Laboratory Science, College of Health Science and Technology, Shanghai Jiao Tong University School of Medicine, Shanghai, China; 6 Central Laboratory of Shanghai Eighth People’s Hospital, Xuhui Branch of Shanghai Sixth People’s Hospital, Caobao Road 8, Shanghai, 200235 China

**Keywords:** Renalase, Obstructive sleep apnea, Hypertension, Single nucleotide polymorphism.

## Abstract

Obstructive sleep apnea (OSA), a condition often linked with hypertension, has an undefined relationship with renalase, a protein known for regulating blood pressure. This study aimed to investigate the relationship between serum renalase levels as well as renalase functional single nucleotide polymorphism (SNP) rs2296545 variant and hypertension in a Han Chinese OSA population. 126 subjects underwent serum renalase detection, with linear regression being performed to evaluate the relationship between serum renalase levels and OSA-related traits. Additional 4275 subjects were obtained rs2296545 genotype information by SNP microarray. And binary logistic regression was used to assess the effect of rs2296545 on hypertension risk. Molecular dynamics simulation and molecular docking were utilized to access the protein structures and the interplay between protein and catecholamines of wild-type and rs2296545 mutant renalase. The results showed that serum renalase levels were significantly higher in the severe OSA group. Further analysis showed renalase levels were positively correlated with blood pressure in the non-OSA group and negatively correlated in the severe OSA group. For rs2296545 polymorphism analysis, the hypertension risk significantly increased for the recessive model CC/GG + CG (OR = 1.211, 95% CI: 1.025–1.431) and the additive model CC/CG (OR = 1.223, 95% CI: 1.025–1.458) in the severe OSA. The rs2296545 polymorphism affected protein structure, and led to increase binding free energy, weakening interactions between renalase and catecholamines. In conclusion, serum renalase levels had independent association with blood pressure. And rs2296545 polymorphism may influence on susceptibility to hypertension by altering protein ability to bind to catecholamines, which might contribute to the intervention of hypertension in the OSA population.

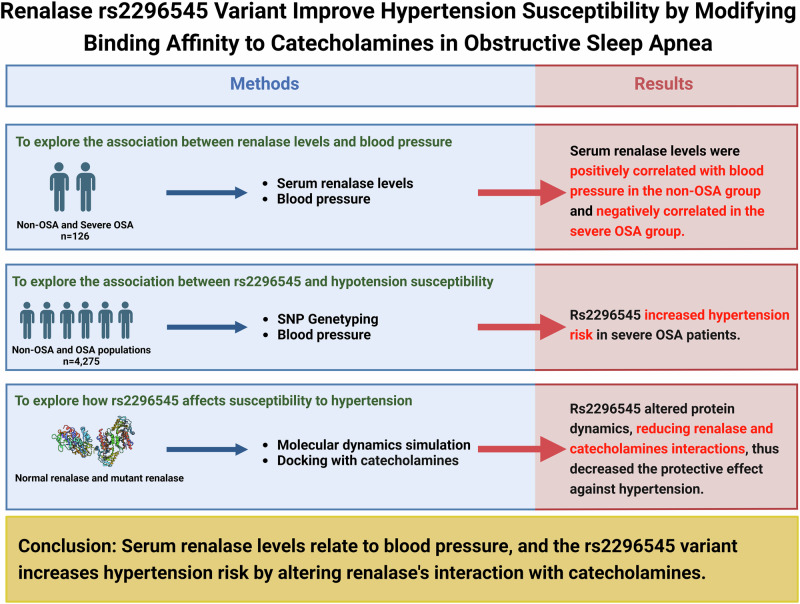

## Introduction

Obstructive sleep apnea (OSA) is a prevalent sleep disorder distinguished by repetitive episodes of upper airway collapse during sleep, resulting in chronic intermittent hypoxia and fragmented sleep [1]. Multiple large-scale epidemiological studies have yielded compelling evidence that apnea or hypopnea function as independent risk factors for hypertension and coronary artery disease [2–4]. Approximately 80% of individuals diagnosed with resistant hypertension exhibit co-occurring OSA, with approximately two-thirds of this patient population ascribing their hypertension primarily to the presence of OSA [5].

Renalase, a flavin adenine dinucleotide-dependent amine oxidase is actively secreted into the bloodstream by the kidneys [6]. Its primary function involves the metabolism of circulating catecholamines such as epinephrine, resulting in the downregulation of cardiac contractility, heart rate, and blood pressure [6–8]. Animal studies have demonstrated that a deficiency in renalase can lead to elevated blood pressure [9], while the introduction of recombinant renalase exhibits a blood pressure-lowering effect [7, 10]. Further, a study found a direct correlation between serum renalase levels, blood pressure, and enhanced hypertension risk [11].

Furthermore, numerous single nucleotide polymorphisms (SNPs) reside within the RNLS gene with certain ones - rs792205, rs10887800, rs2296545, rs2576178, and rs1935582 - reportedly being linked with elevated levels of blood pressure or risk of hypertension [12, 13]. Intriguingly, rs2296545(Asp37Glu) is the exclusive missense mutation among them, leading to an amino acid substitution and potentially impacting the functionality of the gene product [14]. Numerous studies have demonstrated a robust correlation between serum renalase levels and the genetic variant rs2296545 with hypertension and cardiovascular morbidities, including essential hypertension, coronary heart disease, and stroke [12–16].

OSA has a high prevalence of co-morbidity with hypertension, but the mechanisms involved remain unclear. Existing research on the hypoxic disease model of OSA validates the connection between renalase and the apnea-hypopnea index (AHI) yet exhibits limited scale and overlooks the relationship between renalase and blood pressure in OSA patients [17, 18]. Moreover, studies into the correlation between SNPs and hypertension are sparse within OSA [19]. In light of the dearth of data concerning the influence of RNLS variants on hypertension amongst OSA patients, our research set out to explore the association between RNLS gene and rs2296545, and hypertension, along with OSA-related clinical traits in a Han Chinese OSA population. Given the existing evidence indicating a higher prevalence of OSA in males compared to females in the general population, characterized by a male-to-female ratio of approximately 2:1, the participants enrolled in this study exclusively comprised males [1].

## Materials and methods

### Subjects

Suspected OSA patients were identified from the Shanghai Sleep Health Study (SSHS), which is described in our previous study [20]. Anthropometric, biochemical, and polysomnography (PSG) data were meticulously recorded. Genotype information was obtained through the utilization of an SNP microarray (SNP Array 6.0; Affymetrix Inc., San Francisco, CA, USA). Exclusion criteria encompassed individuals aged ≤ 18 years, female sex, individuals previously diagnosed with OSA and treated with positive airway pressure ventilation (CPAP), oral orthotics, or upper airway surgery, instances of missing SNP, systolic blood pressure (SBP), or breathing data, the presence of significant systemic ailments (such as chronic liver disease, renal insufficiency, hyperthyroidism, hypothyroidism, or tumors), the presence of other sleep disorders (such as restless legs syndrome or narcolepsy), and the presence of mental or neurological disorders. Ultimately, a total of 4275 subjects were included for analysis. And additional 126 subjects with matched age and gender underwent serum renalase detection. This study was approved by the Ethics Committee of Shanghai Jiao Tong University Affiliated Sixth People’s Hospital, and informed consent was obtained from all participants. The study was conducted in accordance with the principles outlined in the Declaration of Helsinki.

### Anthropometric and renalase measurement

Body mass index (BMI) was computed by dividing weight (in kilograms) by the square of height (in meters). Blood pressure measurements were obtained on the right arm using an automated electronic device (Omron Model HEM-752 Fuzzy, Omron Corporation), with three consecutive readings taken after a 10-minute period of rest in a seated position, and subsequently averaged. Hypertension was defined as systolic blood pressure (SBP) ≥ 140 mmHg or diastolic blood pressure (DBP) ≥ 90 mmHg, or the utilization of anti-hypertensive medication.

### Polysomnography

Breath-related parameters during sleep were assessed utilizing standard overnight polysomnography (PSG) in accordance with the 2012 recommendations established by the American Academy of Sleep Medicine (AASM) [21]. PSG recordings were conducted using Alice 4 or 5 devices manufactured by Respironics Inc. (Pittsburgh, PA, USA). The apnea-hypopnea index (AHI) was derived by calculating the average number of apnea and hypopnea episodes per hour of sleep. Classification of non-OSA, mild, moderate, and severe OSA was based on AHI thresholds: AHI < 5, 5–15, 15–30, and ≥30 events/h, respectively. The oxygen desaturation index (ODI) was computed by determining the average number of oxygen desaturation episodes ≥3% per hour of sleep, while the micro-arousal index (MAI) was determined by calculating the average number of arousals per hour of sleep.

### SNP genotyping

An analysis was performed extracting information on rs2296545 from the genomic database, with the database description available in the already published GWAS article by our research group [20]. The rs2296545 met the following criteria: a minor allele frequency of less than 0.5 and a Hardy-Weinberg equilibrium test value of *P* > 0.05.

### Serum renalase levels

Serum renalase levels were measured using Enzyme-linked immunosorbent assay (ELISA) kits (Lifespan bioscience, Seattle, WA, USA) were employed to measure serum levels of renalase. according to the manufacturer’s instructions, the assay exhibits a sensitivity of 0.156 µg/ml for human renalase, with an intra-assay coefficient of variation (CV%) < 8% and inter-assay CV% <10%.

### Molecular dynamics simulation

Both wild-type renalase and mutant renalase were modeled using all-atom explicit solvent models in the GROMACS 5.0.6 software. To ensure comparability, both systems utilized identical parameter selection methods, which included a temperature of 310 K and a run time of 50 ns. The force field was set as AMBER99SB, the water model as TIP3P, and the C and N termini were assigned as COO^-^ and NH^3 +^, respectively. A minimum distance of 1 nm was maintained between the protein and the system box. Additional Cl^-^ ions were introduced to neutralize the overall system. Periodic boundary conditions were applied throughout all simulations.

The neutralized system underwent energy minimization employing the steepest descent minimization algorithm. Subsequently, the system was equilibrated under NVT (Number of particles, Volume, and Temperature) conditions for 500 ps and NPT (Number of particles, Pressure, and Temperature) conditions for 1 ns. Pressure parameters were set using the Parrinello-Rahman method to maintain a pressure of 1 bar. Temperature parameters were coupled to an external heat bath with a velocity rescale coupling method to maintain a steady temperature of 310 K. The neighbor list was updated every 10 steps, with a 1.0 nm cutoff distance using the Verlet buffer. All bond lengths were restrained using the LINCS method (for proteins) and the SETTLE algorithm (for water molecules), with an integration time of 2 fs. The cutoff value for van der Waals interaction was set at 1.0 nm. The electrostatic interaction was computed using the particle mesh Ewald (PME) method, with a real space cutoff value set at 1.0 nm. For the statistical analyses, each molecular dynamics trajectory has been repeated 10 times with the same starting structure and different atom velocities.

The GROMACS 5.0.6 package and Python (utilizing the Pandas module) were utilized to calculate the root-mean square deviation (RMSD), root-mean square fluctuations (RMSF) of Cα-atoms, and the gyration of the protein. Visualization of the 3D structures was accomplished using VMD and Pymol.

### Molecular docking

AutoDock free open-source software was run using the AlphaFold predictions of renalase (Glu37) (PDB ID: 3QJ4, homo sapiens) and renalase (Asp37, the substitution of Glu37 with Asp) from UniProt (http://www.uniprot.org), and a chemical table file (Adrenaline, CID: 5816; Norepinephrine, CID: 439260; Isoproterenol, CID: 3779; Dopamine, CID: 681; Dobutamine, CID: 36811) from PubChem (NCBI), with the following parameters: Lamarckian genetic algorithm runs, 300; the maximum number of energy evaluations, 30 million; and grid map setting points and spacing, 126 × 126 × 126 and 1 Å, respectively, in all dimensions [22]. The top candidate docking model was used in this study. Additionally, we verified and visualized three-dimensional protein–ligand complexes using BIOVIA Discovery Studio (Dassault Systèmes, Release 2022), PLIP [23], Protein Plus [24], and PyMOL 2.5.2; the two-dimensional diagrams of these complexes were generated by LIGPLOT +  [25].

### Cavities of the renalase

By uploading the average conformation PDB coordinate files of the wild-type and mutant renalase from two MD simulation systems to the CavityPlus server (http://www.pkumdl.cn:8000/cavityplus2018/), possible cavities of the enzyme were identified [26]. Cavities were generated for the enzyme when no ligand was bound, and the volume size of the cavities was calculated.

### Statistical analysis

Statistical analysis was conducted using IBM SPSS Statistics software (version 26.0, IBM Corp., Armonk, NY, USA). Continuous variables were expressed as mean ± standard deviation, while categorical variables were presented as number and percentage. Prior to the association analysis, the SNP rs2296545 underwent the Hardy-Weinberg equilibrium test using the PLINK software (available at https://zzz.bwh.harvard.edu/plink/data.shtml).

For datasets with a sample size greater than 50, the Kolmogorov-Smirnov test was used to determine if the data is normally distributed. Otherwise, the Shapiro-Wilk test was used for the normality test. Clinical characteristics among groups were compared using t-tests or ANOVA for normally distributed data, and Mann-Whitney U tests or Kruskal-Wallis tests for non-normally distributed data. Sperman’s correlation analysis was employed to investigate the relationship between serum renalase levels and clinical characteristics. The genotype distribution and allele frequencies were compared using the chi-square (χ^2^) test. Three genetic hereditary models were investigated. The dominant model compared the CC + CG genotype with the GG genotype, the recessive model compared the CC genotype with the CG + GG genotype, and the additive model compared the CC genotype with the CG genotype, the CC genotype with the GG genotype, and the CG genotype with the GG genotype, respectively. Logistic regression analysis was employed to evaluate the association between the SNP rs2296545 genetic hereditary models and the risk of hypertension. After adjusting for age, BMI, FPG, TC, TG, HDL, LDL and LPa, odds ratios (OR) with corresponding 95% confidence intervals (CI) were calculated. A two-tailed *P* value < 0.05 was considered statistically significant in the analyses.

## Results

### Baseline characteristics

**Fig. 1:**
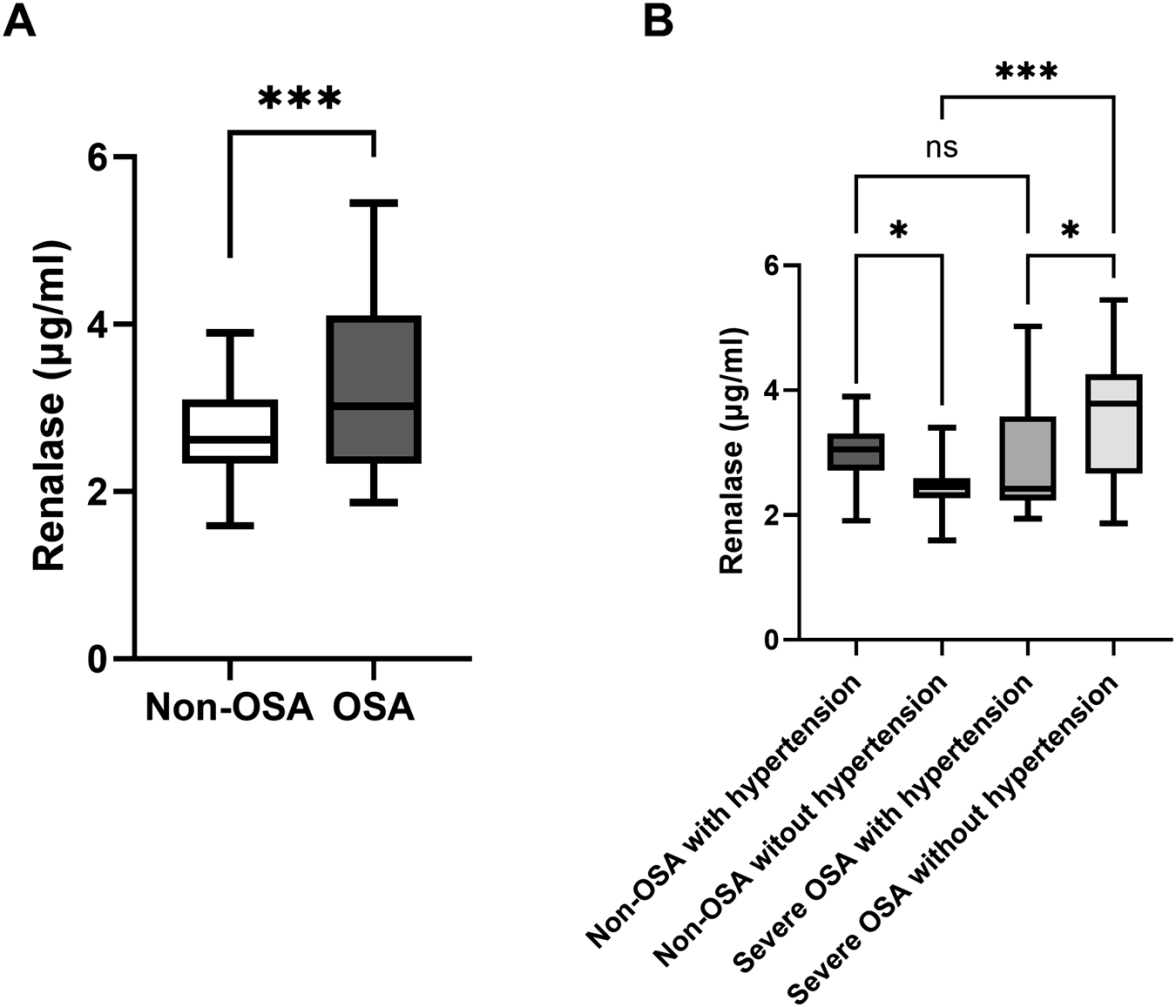
Correlation of serum renalase levels with hypertension and OSA. **A** Serum renalase levels were significantly higher in the OSA population compared to the non-OSA population. **B** Serum renalase levels are elevated when patients have hypertension or OSA. In the non-OSA group, serum renalase levels were seen to be elevated in the hypertension subgroup compared to the non-hypertension group. In the severe OSA group, compared to the non-hypertension subgroup, the serum renalase levels in the hypertension subgroup decreased. ns: no significance; *p < 0.05; **p < 0.01; ***p < 0.001

Based on the standard of OSA severity, the population tested for renalase serum levels which consisted of 64 non-OSA and 62 severe OSA patients, with no statistically significant difference in age and BP, and a higher indicators of glycolipid metabolism and breath related parameters in patients with severe OSA (*p* < 0.05, Table 1). Then we also sub-grouped the matched group by hypertension (with or without) and found there no significant differences in indicators of glycolipid metabolism and breath related parameters (*p* > 0.05, Table 1). Serum renalase levels were significantly higher in the OSA group compared to the non-OSA group (3.26 ± 0.82 μg/ml vs 2.72 ± 0.25 μg/ml, *p* = 0.0003). Specifically, in the non-OSA group with hypertension, the serum renalase levels exhibited a significant elevation in comparison to the non-OSA group without hypertension (2.98 ± 0.84 μg/ml vs 2.45 ± 0.32 μg/ml, *p* = 0.011). Conversely, within the severe OSA group, the serum renalase levels in individuals with hypertension displayed a significant decrease when contrasted with those in individuals without hypertension (2.94 ± 0.91 μg/ml vs 3.57 ± 1.05 μg/ml, *p* = 0.027). Additionally, a notable disparity was observed between the serum renalase levels in the non-OSA group without hypertension and the OSA group without hypertension, with the former displaying significantly lower levels (2.45 ± 0.32 μg/ml vs 3.57 ± 1.05 μg/ml, *p* < 0.0001). But no significant distinction in serum renalase levels was detected between the non-OSA group with hypertension and the severe OSA group with hypertension (2.98 ± 0.84 μg/ml vs 2.94 ± 0.91 μg/ml, *p* = 0.617). More intuitive comparative analysis of renalase levels is shown in Fig. 1. Furthermore, a substantial correlation was observed between serum renalase levels and parameters such as AHI, MAI and ODI (β = 0.27, *p* = 0.002, β = 0.203, *p* = 0.023, β = 0.265, *p* = 0.003, respectively), even adjusting for age and BMI (β = 0.242, *p* = 0.005; β = 0.189, *p* = 0.035; β = 0.232, *p* = 0.007, respectively). As for SBP and DBP, more specifically, in the non-OSA group, there was a positive correlation with SBP and DBP when adjusting for age and BMI (β = 0.488, *p* < 0.001; β = 0.398, *p* = 0.002, respectively). In contrast, a negative correlation was noted amongst individuals with severe OSA adjusting for age and BMI (β = −0.307, *p* = 0.026; β = −0.345, *p* = 0.012, respectively), as elaborated in Table S1.

**Table 1:** Clinical characteristics of the 126 subjects with measured serum renalase levels

Traits	Non-OSA with hypertension (*n* = 31)	Non-OSA without hypertension (*n* = 33)	*p*^a^	Severe OSA with hypertension (*n* = 31)	Severe OSA without hypertension (*n* = 31)	*p*^b^	Total (*n* = 126)	*p*^c^
AHI	2.60 (1.30–4.00)	2.50 (1.80–3.70)	0.936	52.40 (37.00–68.95)	53.10 (31.15–63.10)	0.531	4.85 (2.52–52.27)	**<.001**
Age (years)	36.00 (31.00–45.00)	37.00 (31.00–48.00)	0.545	36.00 (31.50–47.00)	37.00 (32.50–47.50)	0.583	37.00 (31.00–47.00)	0.857
BMI (kg/m2)	27.68 (24.22–28.04)	24.22 (21.97–25.99)	0.007	27.36 (25.63–28.56)	25.95 (23.97–27.61)	0.112	25.95 (24.10–27.78)	**<.001**
FPG (mmol/L)	5.31 (5.02–5.68)	5.24 (4.96–5.60)	0.582	5.49 (5.10–5.71)	5.26 (5.06–5.54)	0.364	5.29 (5.01–5.65)	0.715
TC (mmol/L)	4.45 (4.08–5.03)	4.54 (3.85–4.77)	0.936	4.66 (4.43–5.58)	4.32 (3.94–4.83)	0.038	4.50 (4.04–5.12)	0.09
TG (mmol/L)	1.69 (1.25–2.21)	1.21 (0.74–1.82)	0.048	1.71 (1.33–2.78)	1.61 (1.45–2.13)	0.688	1.60 (1.15–2.22)	0.063
HDL-C (mmol/L)	1.00 (0.86–1.12)	1.07 (0.94–1.18)	0.166	1.01 (0.92–1.08)	1.02 (0.92–1.19)	0.8	1.02 (0.91–1.15)	0.571
LDL-C (mmol/L)	2.70 (2.50–3.25)	2.48 (2.22–3.22)	0.497	2.92 (2.69–3.68)	2.74 (2.35–3.29)	0.055	2.76 (2.36–3.34)	0.059
SBP (mmHg)	142.00 (140.00–150.00)	120.00 (111.00–121.00)	**<.001**	140.00 (137.00–150.00)	120.00 (112.00–124.00)	**<.001**	129.00 (120.00–141.00)	**<.001**
DBP (mmHg)	95.00 (87.50–99.50)	76.00 (71.00–80.00)	**<.001**	96.00 (89.50–102.00)	79.00 (74.50–80.00)	**<.001**	84.00 (78.00–95.00)	**<.001**
Renalase (μg/ml)	3.05 (2.79–3.29)	2.45 (2.27–2.57)	**<.001**	2.42 (2.27–3.48)	3.79 (2.67–4.23)	**0.014**	2.72 (2.34–3.37)	**<.001**
Insulin (μU/mL)	10.61 (6.64–16.42)	6.38 (4.20–11.21)	**0.029**	11.71 (10.57–19.35)	12.75 (8.93–17.16)	0.773	11.09 (6.46–16.61)	**0.001**
ApoA- I (g/L)	1.00 (0.90–1.10)	0.98 (0.89–1.09)	0.994	0.93 (0.84–1.04)	0.95 (0.85–1.03)	0.806	0.96 (0.86–1.09)	0.542
ApoB (g/L)	0.83 (0.72–0.94)	0.84 (0.65–0.95)	0.96	0.93 (0.82–1.14)	0.90 (0.72–0.96)	0.096	0.89 (0.73–0.99)	**0.019**
ApoE (mg/dL)	3.92 (3.46–4.65)	3.31 (2.86–4.58)	0.276	4.49 (3.09–5.18)	3.94 (3.45–4.71)	0.554	3.94 (3.09–4.84)	0.364
HOMA-IR	2.65 (1.60–3.69)	1.52 (0.93–2.67)	0.037	2.99 (2.21–4.65)	3.04 (2.10–4.41)	0.685	2.60 (1.55–4.13)	**0.004**
NC (cm)	38.25 (36.25–39.75)	39.00 (38.00–40.00)	0.406	40.00 (39.00–42.25)	41.00 (39.00–42.00)	0.63	40.00 (38.00–42.00)	0.055
WC (cm)	93.00 (85.75–97.00)	90.00 (85.00–93.00)	0.368	97.00 (94.50–101.50)	96.00 (89.50–102.00)	0.421	96.00 (90.00–101.20)	**0.007**
HC (cm)	99.50 (94.00–104.00)	98.50 (94.00–102.00)	0.559	103.00 (100.50–107.00)	100.00 (99.00–105.00)	0.061	101.00 (99.00–105.00)	**0.009**
WHR	0.93 (0.92–0.95)	0.91 (0.89–0.95)	0.382	0.95 (0.91–0.97)	0.96 (0.91–1.00)	0.588	0.94 (0.91–0.97)	0.23
ESS	5.50 (3.00–7.50)	1.00 (0.00–7.00)	0.269	8.00 (7.00–11.00)	9.00 (5.50–13.50)	0.528	8.00 (5.00–11.50)	**0.005**
MAI	13.40 (9.70–21.95)	12.20 (8.50–22.80)	0.653	31.40 (16.40–50.05)	24.00 (12.65–39.70)	0.205	17.80 (10.60–32.20)	**<.001**
Minimum SaO_2_(%)	91.00 (89.75–92.25)	89.00 (88.00–93.00)	0.401	74.00 (59.75–79.00)	73.00 (67.00–78.00)	0.665	78.00 (69.25–87.75)	**<.001**
ODI	1.90 (1.15–3.55)	2.70 (1.80–3.70)	0.317	52.60 (35.60–69.25)	51.90 (29.15–62.70)	0.588	12.35 (2.32–51.57)	**<.001**

Bold values are statistically significant (*p* < 0.05). p^a^ for non-OSA without hypertension venous non-OSA with hypertension; p^b^ for severe OSA without hypertension venous severe OSA with hypertension; p^c^ for differences between the five groups

*OSA* obstructive sleep apnea, *AHI* apnea–hypopnea index, *BMI* body mass index, *FPG* fasting plasma glucose, *TC* total cholesterol, *TG* triglyceride, *HDL-C* high-density lipoprotein cholesterol, *LDL-C* low-density lipoprotein cholesterol, *SBP* systolic blood pressure, *DBP* diastolic blood pressure, *ApoA-I* apolipoprotein A-I, *ApoB* apolipoprotein B, *ApoE* apolipoprotein E, *HOMA-IR* homeostatic model assessment for insulin resistance, *NC* neck circumference, *WC* waist circumference, *HC* hip circumference, *WHR* waist/hip circumference ratio, *ESS* Epworth Sleepiness Scale, *SaO2* oxygen saturation, *ODI* oxygen desaturation index

Similarly, based on the standard of OSA severity, the genotyping population included 629 non-OSA (166 hypertensions, 483 non-hypertensions), 280 mild OSA (71 hypertensions, 209 non-hypertensions), 857 moderate OSA (384 hypertensions, 473 non-hypertensions) and 2509 severe OSA patients (1470 hypertensions, 1039 non-hypertensions). Patients with OSA were predominantly older, more obese, a had higher blood pressure (p < 0.05, Table 2). As OSA severity increased, an observable escalation in the prevalence of hypertension emerged, registering rates of 26.39%, 25.36%, 44.81%, and 58.59% sequentially (p < 0.001). Moreover, the distribution of the genotypes did not exhibit any considerable difference within the stratified subgroups (*p* = 0.741).

**Table 2:** Clinical characteristics of the overall population classified by the severity of OSA

Traits	non (*n* = 629)	mild (*n* = 280)	moderate (*n* = 857)	severe (*n* = 2509)	Total (*n* = 4275)	*p*
Age (years)	37.70 ± 11.66	32.41 ± 4.89	43.44 ± 12.39	43.61 ± 11.54	41.98 ± 11.88	**<0.001**
AHI	2.25 ± 1.40	10.70 ± 2.32	22.12 ± 4.35	58.58 ± 16.71	39.85 ± 26.48	**<0.001**
BMI (kg/m^2^)	24.37 ± 3.55	25.27 ± 2.98	26.77 ± 8.71	28.22 ± 4.46	27.17 ± 5.60	**<0.001**
FPG (mmol/L)	5.24 ± 0.96	5.07 ± 0.45	5.49 ± 1.18	5.72 ± 1.26	5.56 ± 1.19	**<0.001**
TC (mmol/L)	4.45 ± 0.87	4.62 ± 0.89	4.76 ± 0.91	4.87 ± 0.95	4.77 ± 0.94	**<0.001**
TG (mmol/L)	1.69 ± 1.59	1.72 ± 1.72	2.03 ± 1.62	2.36 ± 1.98	2.15 ± 1.86	**<0.001**
HDL-C (mmol/L)	1.07 ± 0.26	1.07 ± 0.23	1.02 ± 0.23	1.01 ± 0.22	1.02 ± 0.23	**<0.001**
LDL-C (mmol/L)	2.72 ± 0.73	2.94 ± 0.80	2.99 ± 0.79	3.03 ± 0.81	2.97 ± 0.80	**<.001**
ApoA (g/L)	1.05 ± 0.19	1.05 ± 0.17	1.05 ± 0.19	1.06 ± 0.18	1.06 ± 0.18	0.396
ApoB (g/L)	0.78 ± 0.21	0.82 ± 0.18	0.86 ± 0.19	0.89 ± 0.20	0.86 ± 0.20	**<0.001**
ApoE (mg/dL)	4.13 ± 1.63	4.15 ± 1.50	4.60 ± 1.89	5.01 ± 2.17	4.74 ± 2.04	**<0.001**
LPa	12.80 ± 14.50	13.23 ± 14.70	12.25 ± 14.04	11.17 ± 12.92	11.76 ± 13.53	0.005
Insulin (μU/mL)	9.35 ± 7.81	11.14 ± 6.43	12.58 ± 8.65	15.54 ± 10.30	13.75 ± 9.72	**<0.001**
SBP (mmHg)	121.46 ± 13.45	124.01 ± 13.45	126.70 ± 15.97	129.62 ± 15.74	127.47 ± 15.62	**<0.001**
DBP (mmHg)	77.88 ± 9.93	78.91 ± 9.96	80.53 ± 10.99	83.97 ± 11.91	82.05 ± 11.59	**<0.001**
MAI	18.05 ± 14.36	21.52 ± 15.63	24.31 ± 17.07	39.09 ± 22.88	31.88 ± 22.11	**<0.001**
Minimum SaO_2_	89.93 ± 11.81	86.03 ± 6.48	81.09 ± 8.92	69.10 ± 12.25	75.68 ± 13.98	**<0.001**
Average SO_2_	95.95 ± 3.92	95.63 ± 1.58	94.88 ± 1.80	91.83 ± 3.78	93.30 ± 3.82	**<0.001**
ODI	4.61 ± 12.61	11.14 ± 6.23	23.23 ± 10.22	58.50 ± 20.38	40.40 ± 28.05	**<0.001**
CT90	0.73 ± 5.78	1.20 ± 4.43	3.97 ± 7.25	21.27 ± 18.56	13.46 ± 17.51	**<0.001**
Hypertension, *n* (%)						**<0.001**
non-hypertension	483 (73.61)	209 (74.64)	473 (55.19)	1039 (41.41)	2184 (51.09)	
hypertension	166 (26.39)	71 (25.36)	384 (44.81)	1470 (58.59)	2091 (48.91)	
Rs2296545, *n* (%)						0.741
CC	216 (34.34)	105 (37.50)	293 (34.19)	894 (35.63)	1508 (35.27)	
CG	300 (47.69)	134 (47.86)	409 (47.72)	1207 (48.11)	2050 (47.95)	
GG	113 (17.97)	41 (14.64)	155 (18.09)	408 (16.26)	717 (16.77)	

Bold values are statistically significant (*p* < 0.05)

*OSA* obstructive sleep apnea, *AHI* apnea–hypopnea index, *BMI* body mass index, *FPG* fasting plasma glucose, *TC* total cholesterol, *TG* triglyceride, *HDL-C* high-density lipoprotein cholesterol, *LDL-C* low-density lipoprotein cholesterol, *ApoA-I* apolipoprotein A-I, *ApoB* apolipoprotein B, *ApoE* apolipoprotein E, *SaO2* oxygen saturation, *ODI* oxygen desaturation index

### Association of rs2296545 polymorphism and risk of hypertension

In our population, G and C was the minor and major allele of rs2296545 polymorphism respectively. The genotype distribution did not deviate significantly from Hardy–Weinberg equilibrium test (*p* > 0.05), suggesting the alleles were under equilibrium.

We investigated the association between rs2296545 and indicators of glycolipid metabolism, blood pressure and breath related parameters including respiratory events and oxygen traits. We observed no substantial correlation between the SNPs and quantitative traits across all the participants, as detailed in Table S2. We analyzed the alleles and genotypes of rs2296545 polymorphism in the hypertension and non-hypertension groups using additive, dominant, and recessive models (Table 3 and Table S3). In the severe OSA subgroup, the hypertensive disease risk significantly increases for the recessive model genotype CC/GG + CG after adjusting for age, BMI, FPG, TC, TG, HDL, LDL and LPa (OR = 1.270, 95% CI: 1.068–1.512, *p* = 0.0071). In other subgroup analyses, no discernible relationship between the identified genotypes and the risk of hypertension is observed.

**Table 3:** Association of rs2296545 polymorphism with the hypertension risk in recessive model

	Non OSA (*n* = 629)		Mild OSA (*n* = 280)		Moderate OSA (*n* = 857)		Severe OSA (*n* = 2509)		OSA (*n* = 3646)		Total (*n* = 4275)	
	OR (95%CI)	*p*	OR (95%CI)	*p*	OR (95%CI)	*p*	OR (95%CI)	*p*	OR (95%CI)	*p*	OR (95%CI)	*p*
Recessive model												
CC/GG + CG	0.975 (0.673–1.412)	0.892	1.414 (0.817–2.446)	0.215	0.799 (0.601–1.062)	0.122	1.211 (1.025–1.431)	0.025	1.099 (0.959–1.260)	0.173	1.088 (0.959–1.233)	0.19
CC/GG + CG*	0.894 (0.598–1.325)	0.580	1.442 (0.819–2.531)	0.203	0.854 (0.638–1.142)	0.288	1.27 (1.068–1.512)	**0.0071**	1.143 (0.992–1.317)	0.064	1.115 (0.978–1.273)	0.105

Because of insignificant associations under the dominant and additive models, only the recessive model is listed. The ORs with 95% CIs are shown for corresponding genotypes. Binary logistic regression was performed using a proportional odds model that passed the parallel lines test (*p* > 0.05). Bold values are statistically significant (p < 0.05). Models* were adjusted for age, BMI, FPG, TC, TG, HDL, LDL and LPa. OR, Odds ratio; CI, Confidence interval. Additive, dominant and recessive models were based on minor allele of each locus

### Molecular dynamics simulation of rs2296545 of renalase

**Fig. 2:**
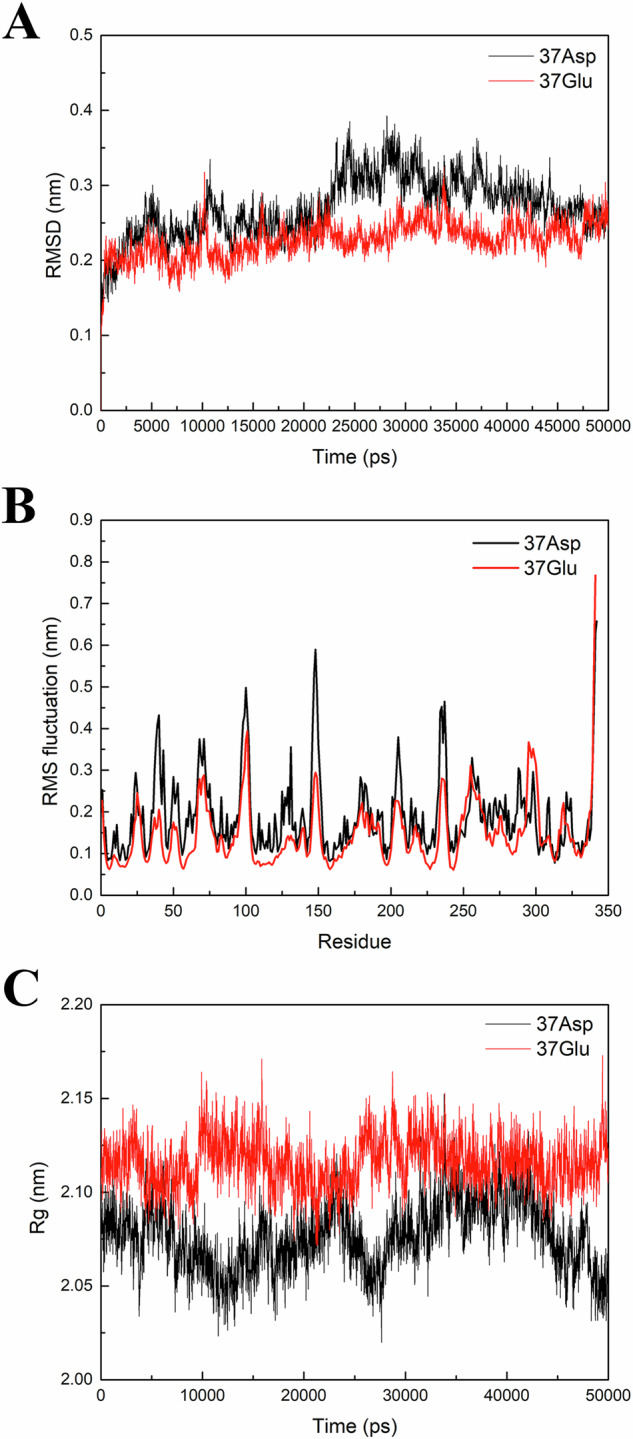
**A** The RMSD of renalase(37Glu) stabilizes faster compared to renalase(37Asp). **B** The structural integrity of the renalase protein conformation undergoes perturbation, resulting in increased flexibility in renalase(Asp37). **C** The increased density in renalase (37Asp) may reduce the enzymatic activity pocket, impacting renalase substrate binding

In our investigation, subsequent to simulation, we embarked on a comprehensive analysis of RMSD to scrutinize the dynamic alterations transpiring between the protein backbone and its initial conformation. As shown as Fig. 2A, during the simulation, the RMSD of residue 37Glu swiftly attained a steady state, converging at an approximate value of 0.23 nm. Conversely, the RMSD of residue 37Asp exhibited marked relative fluctuations, persistently escalating within the initial 25 ns of the simulation. Only after a simulation duration of 28 ns did the RMSD value of residue 37Asp commence stabilization, implying a greater propensity for conformational diversity relative to 37Glu within the aqueous medium. This intriguing observation intimates a plausible association between the fidelity of the initial model and the augmented structural flexibility arising from the single-point SNP in residue 37Asp.

RMSF serve as a valuable quantitative measure to elucidate the dynamic behavior of amino acid residues throughout the simulation, providing insights into the inherent flexibility of proteins. Notably, the substitution of Glu37 with Asp induces a discernible augmentation in loop dynamics across the entire protein, particularly in the vicinity of the loop region (residues 34–40) encompassing the Glu37Asp mutation site. Through meticulous RMSF analysis, it is revealed that the serine residue at position 148 (148Thr) within the renalase(Asp37) exhibits the most pronounced root-mean-square fluctuation, measuring approximately 0.59 nm. This value surpasses the corresponding RMSF of 0.3 nm observed in the renalase(Glu37) at the identical 148Thr position. More details are shown in the Fig. 2B. Consequently, the stability of the protein conformation in the Asp37-mutated enzyme becomes compromised, leading to an elevated degree of flexibility.

The Radius of Gyration (RG) analysis was performed on the protein simulation results. RG is the root mean square distance of all masses from their center of mass, providing an “equivalent distance” between mass and the rotational axis. It can be used to represent the compactness of a protein, considering the area (S) of its cross-sectional shape. From the Fig. 2C, it is evident that the overall RG of renalase(37Glu) remains relatively stable, while the RG of renalase(37Asp) exhibits more pronounced fluctuations, with a peak value of 2.15 nm and a minimum value of 2.08 nm. Analysis of the RG data suggests a denser protein structure for renalase(37Asp), potentially leading to a smaller active pocket and influencing the binding of renalase with substrates.

To explore this further, the average conformations were extracted from the 50-nanosecond dynamic simulation trajectories of both renalase(37Asp) and renalase(37Glu). The volume of the substrate-binding pocket was then calculated for each variant. The pocket volume of the renalase(37Asp) and substrate complex was determined to be 1565.95 Å³, whereas the renalase(37Glu) and substrate complex exhibited a larger pocket volume of 1940.54 Å³, clearly surpassing that of renalase(37Asp), seeing Fig. S1 for details.

### Renalase docking with catecholamines

**Fig. 3:**
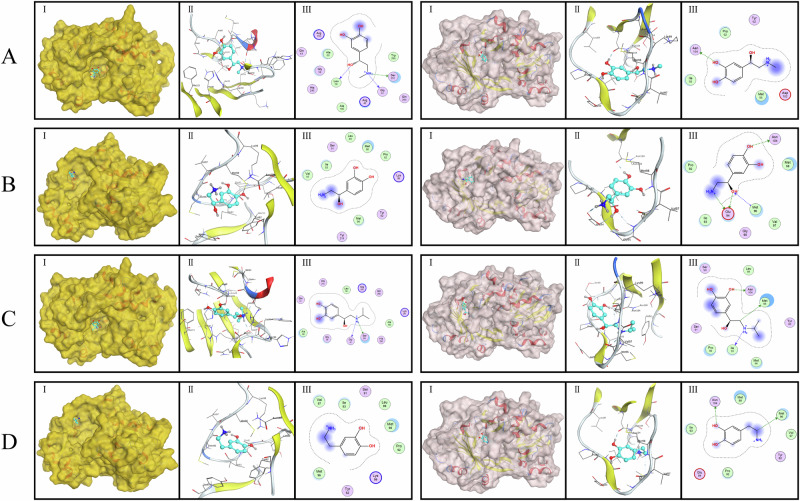
Renalase (37Glu) on the left and renalase (37Asp) on the right. **A** Adrenaline; (**B**): norepinephrine; (**C**): isoprenaline; (**D**): dopamine. I: Binding mode of wild-type or mutant renalase proteins to individual catecholamines; II: Three-dimensional interactions of renalase proteins and catecholamines; III: Two-dimensional interactions of renalase proteins and catecholamines

We conducted protein-ligand docking simulations to investigate the interaction between two simulated models of renalase protein and a range of catecholamines, as illustrated in Fig. 3. Subsequently, we computed the binding free energies of these complexes, which characterize the interaction between a ligand and its receptor. A lower binding free energy suggests a tighter interaction between the protein and ligand, and typically indicates a more stable binding with higher biological activity. Conversely, a higher binding free energy signifies a weaker interaction between the protein and ligand, which may imply a less stable binding with reduced or negligible biological activity.

Remarkably, the Glu mutation at position 37 to Asp (rs2296545) induced an elevation in the binding free energy between renalase and catecholamines. Specifically, the binding free energy with adrenaline increased from −5.5054 kcal/mol to −5.0347 kcal/mol, with norepinephrine from −5.2662 kcal/mol to −5.0220 kcal/mol, with isoproterenol from −6.4604 kcal/mol to −5.6194 kcal/mol, with dopamine from −5.2179 kcal/mol to −4.8600 kcal/mol, and with dobutamine from −6.7809 kcal/mol to −6.7701 kcal/mol (Table S4). Upon mutation, renalase displayed the most significant increase in binding free energy to isoprenaline, subsequently followed by epinephrine, dopamine, norepinephrine, and dobutamine in the sequence of descending affinity. These findings underscore a diminished stability in the binding interaction between renalase and catecholamines subsequent to the mutation.

## Discussion

This study establishes a significant link between serum renalase levels and OSA with hypertension that heightened serum renalase levels concentrations in OSA patients compared to the non-OSA cohort. Interestingly, serum renalase levels positively correlates with SBP and DBP in non-OSA individuals, while inversely in severe OSA cases. Renalase SNP rs2296545 variant is notably associated with an increased risk of hypertension in severe OSA individuals. Molecular dynamics and docking simulations of rs2296545 reveal decreased binding affinity between the mutated renalase protein and certain catecholamines, offering a reference for understanding the susceptibility to hypertension in patients, which could guide prevention and treatment strategies.

In this investigation, we assessed serum renalase levels among participants from an independent sample, leading to the identification of a substantial rise in serum renalase levels among individuals afflicted with severe OSA compared to the non-OSA population. Contrarily, in a demographic largely consisting of non-OSA and mild-to-moderate OSA patients, serum renalase levels were reduced [18]. This intriguing contradiction potentially stems from varying degrees of hypoxia among the cases, yet one defining conclusion drawn is that serum renalase levels are subject to hypoxic conditions. Hypoxia presents as a pivotal pathological feature of OSA [27]. Numerous prior studies have demonstrated that serum hypoxia-inducible factor 1α (HIF-1α) protein levels are elevated in patients with OSA and return to normal after two months of Continuous Positive Airway Pressure (CPAP) treatment [28–31]. HIF-1α is a member of a family of transcription factors that are inherently present in cells, sensitive to oxygen levels, and linked to von Hippel-Lindau proteins under normal oxygen conditions, leading to their degradation via the proteasome [32, 33]. Conversely, the degradation of HIF-1α is inhibited in hypoxic conditions [34]. Several studies have shown, renalase under the influence of HIF-1α, demonstrates an increasing trend within hypoxic tissues [35–37]. This observation could potentially provide the underlying explanation for the concomitant rise of renalase within the patient populace of OSA. It has been demonstrated that renalase exhibits a protective effect against H_2_O_2_-induced damage in intestinal cells by mitigating oxidative stress [35, 38]. Meanwhile, several studies have claimed that oxidative stress induced by OSA may be an important etiologic factor that predispose patients with OSA to hypertension [39–43]. Thus, compensatory elevation of renalase could mitigate hypertension in OSA by reducing oxidative stress metabolites and improving blood pressure. Animal studies with renalase knockout mice support this, showing higher catecholamine levels and hypertension symptoms compared to wild-type mice [9]. Hypoxia and oxidative stress could contribute to elevated serum renalase levels in OSA patients.

Specific to subgroups, within the non-OSA group, serum renalase levels were seen to be elevated in the hypertension subgroup when compared to the non-hypertension group. This aligns with a separate study [15], which demonstrated significantly elevated serum renalase levels in hypertensive adolescents compared to their healthy counterparts. Additionally, within the hypertensive group, a positive correlation was observed between serum renalase levels and 24-hour SBP as well as 24-hour DBP [44]. Generally, patients with hypertension exhibit significantly elevated serum catecholamine levels [45–47]. Moreover, individuals diagnosed with both hypertension and OSA display higher catecholamine levels than those with either condition alone [48]. As highlighted previously, renalase acts to neutralize the effects of catecholamines. However, the metabolic capacity of renalase is limited: when the amount of catecholamines is excessive, the protein content and activity of renalase will rapidly decrease [49]. This is also reflected in the severe OSA group, where, compared to the non-hypertension subgroup, the serum renalase levels in the hypertension subgroup decreased. Furthermore, serum renalase levels showed a negative correlation with SBP and DBP. We hypothesize that an initial rise in blood pressure triggers a compensatory response, augmenting renalase. However, this ability has limits; once exceeded, renalase levels decline, contributing to further blood pressure elevation. Further research is needed to clarify renalase compensation mechanisms and its role in blood pressure regulation.

The RNLS gene encompasses numerous SNPs, among which rs2296545 is situated within exon 2, resulting in an amino acid substitution (specifically, the replacement of glutamic acid at codon 37 with aspartic acid, Glu37Asp) [14]. This substitution holds the potential to influence the functional characteristics of the resultant gene product [14, 50]. Previous investigations within the Han Chinese population of northern China have posited a correlation between the rs2296545 CC genotype and essential hypertension, implicating a heightened predisposition to hypertension in individuals bearing this genetic variant [15]. Our findings unveiled a substantial escalation in hypertension risk among severe OSA patients exhibiting the rs2296545 recessive model CC/GG + CG. Moreover, a meta-analysis substantiated a noteworthy correlation between rs2296545 and an elevated susceptibility to hypertension [12]. Beyond its association with hypertension, rs2296545 manifested correlations with various other maladies, encompassing chronic kidney disease [51], diabetic retinopathy [50], myocardial hypertrophy [14], and ischemic stroke [52]. However, no association between rs2296545 and hypertension incidence was found in our OSA cohorts, likely due to the predominance of severe OSA cases. Further exploration in diverse OSA severity populations is necessary. Additionally, no significant disparities in blood pressure levels among different rs2296545 genotypes in the OSA demographic were observed, possibly due to the limited impact of a single SNP on blood pressure. Nevertheless, our study supports that rs2296545 could heighten hypertension susceptibility in OSA patients.

The initiation of hypertension is intricately associated with multiple factors, with catecholamines such as adrenaline, noradrenaline, and dopamine playing pivotal roles in this complex regulatory network. Previous research posits that renalase, through its catecholamine-metabolizing activity, significantly contributes to the modulation of blood pressure [49]. To comprehensively unravel the mechanistic underpinnings of how rs2296545 amplifies susceptibility to hypertension in individuals with OSA, we conducted molecular dynamics simulations of the renalase protein both pre- and post-mutation, complemented by docking simulations involving catecholamine-like molecules. The outcomes of the molecular dynamics simulation elucidated that the Glu37Asp mutation induced alterations in the structural stability of renalase at position 37. These changes resulted in decreased protein stability, heightened flexibility, structural compaction, and a reduction in the size of the enzyme’s substrate-binding cavity. Consequently, the challenge associated with substrate binding was augmented. The docking simulations further substantiated these findings, revealing a diminished binding affinity of renalase (37Asp) for various catecholamine molecules compared to the pre-mutation renalase (37Glu). In a correlated investigation, the authors noted that the Glu37Asp (CC genotype) led to a 24-fold decrease in affinity for NADH and a 2.3-fold reduction in maximum renalase activity [14]. Notably, the rs2296545 codon 37 (Asp37Glu) is situated proximally to the C-terminal of renalase’s FAD binding site, and the enzyme’s oxidase activity is heavily contingent on FAD. The structural alterations ensuing from the mutation are likely the primary determinants of the observed decline in renalase activity. The intricate interplay between rs2296545 and the spatial proximity of the codon 37 mutation to the FAD binding site underscores the potential mechanistic foundation for the diminished renalase activity and its consequential impact on susceptibility to hypertension. We hypothesized that this variation could lead to alterations in enzyme functionality, potentially diminishing catecholamine breakdown and contributing to the onset of hypertension. A prior study demonstrated notably elevated adrenaline levels in CKD patients possessing the rs2296545 CC genotype in contrast to those with the CG and GG genotypes, partially confirming our hypothesis [51].

The mechanisms by which renalase lowers blood pressure can be categorized into three distinct pathways. Initially, it indirectly diminishes blood pressure by metabolizing catecholamines [49, 53]. Furthermore, it regulates blood pressure by inhibiting the activation of the renal dopamine (DA) system [54, 55]. Lastly, it influences blood pressure by attenuating renal sympathetic nerve activity [56, 57]. OSA contributes to hypertension through intermittent hypoxia, which increases sympathetic nerve activity and raises plasma catecholamine levels, leading to vasoconstriction [58–60]. Additionally, hypoxia-induced oxidative stress promotes ROS formation, causing vasoconstriction by impeding NO synthase, increasing endothelin-1 production, and stimulating angiotensin II activation [39, 42, 43, 61]. Renalase also reduces ROS production, providing an antioxidative effect [35, 38]. Consequently, renalase’s antihypertensive effects may be more pronounced in individuals with OSA than those with general hypertension. This could explain the more substantial impact of the rs2296545 variant on hypertension incidence in the severe OSA subgroup in our study.

Our research identifies a significant association between SNP rs2296545 and hypertension risk in the OSA population, offering insights into its pathogenesis and implications for personalized therapies. Genetic information is key to personalized therapy, yet it demands a deep understanding of the complex interplay between various SNPs, genes, and environmental factors. Consequently, thorough validation across independent cohorts is essential to confirm its effectiveness in personalized therapy.

### Perspectives of Asian

In recent years, there has been a noticeable rise in the incidence of OSA in Asia due to shifts in lifestyle, escalating obesity rates, and heightened awareness about OSA. And OSA is intricately linked to hypertension. However, the area of research surrounding renalase and hypertension within populations affected by OSA remains notably underserved, with extant studies often limited by modest sample sizes or lacking in-depth analyzation. This study’s primary strength lies in its pioneering investigation of the relationship between rs2296545 and hypertension in a large sample of individuals with OSA. And the results are consistent with those of several previous studies conducted among different ethnic groups, including Chinese, Egyptian, Polish, and Swedish [11, 15, 16, 62]. Furthermore, the study employed molecular docking and molecular dynamics simulations to preliminarily validate the structural and functional impacts of rs2296545 on the renalase protein at the molecular level. Renalase is expected to contribute to the treatment of hypertension, especially in the OSA population. Future prospective studies are necessary to further validate the association between renalase and hypertensive disorders in Asian populations.

### Limitations

Several limitations of the present study should be addressed. Firstly, all participants included in the study were Han Chinese males, and the OSA patients were predominantly severe cases. Therefore, the generalizability and applicability of our findings may be more pertinent to males with severe OSA. Variations in genetic makeup, environmental exposures, and socioeconomic conditions, often associated with different ethnic groups, can influence study-related variables. Consequently, the applicability of our findings to a broader demographic may be restricted. There is a need to expand the sample to include different genders and multiple ethnicities in the future to improve the generalizability and accuracy of the findings. Secondly, social environmental factors such as diet, exercise, and economic status, which could potentially play a role, were not considered. Lastly, the results validated through simulations often rely on theoretical models and assumed conditions, which may deviate from the complexity found in real biological systems. Thus, while simulations provide a valuable starting point for research, they are not a complete substitute for experimental validation. Future studies should employ advanced techniques like high-performance nuclear magnetic resonance (NMR) spectroscopy or fluorescence resonance energy transfer (FRET) for further validation of our findings from molecular docking simulations.

## Conclusion

In conclusion, this investigation reveals a nuanced interconnection between serum levels of renalase and the co-occurrence of severe OSA with hypertension. Rs2296545 variant may influence on susceptibility to hypertension by altering the ability of renalase to bind to catecholamines. Furthermore, the discernible impact of a renalase, as a promising novel target for both the prophylaxis and intervention of hypertension in the OSA population.

## Supplementary information

Supplementary appendix
