# Supplementary material for: Renalase rs2296545 variant improve hypertension susceptibility by modifying binding affinity to catecholamines in obstructive sleep apnea
Source: Hypertens Res. 2024 Sep 4;47(11):3200–13. doi: 10.1038/s41440-024-01850-0 (PMC11534681; doi:10.1038/s41440-024-01850-0)

**Supplementary appendix**

**Renalase rs2296545 Variant Improve Hypertension Susceptibility by Modifying Binding Affinity to Catecholamines in Obstructive Sleep Apnea**

**Supplementary Table S1.** Association of serum renalase levels with indicators of glycolipid metabolism, blood pressure and sleep-related indicators.

**Supplementary Table S2.** Association of rs296545 with indicators of glycolipid metabolism, blood pressure and sleep-related indicators.

**Supplementary Table S3.** Association of rs2296545 polymorphism with the hypertension risk in dominant and additive genetic models.

**Supplementary Table S4.** Binding energy of renalase (37Glu) and renalase (37Asp) in combination with several catecholamines.

**Supplementary Figure S1.** Rs2296545 makes the mutation of amino acid 37 from Glu to Asp, resulting in a smaller active pocket volume.

**Table S1.** Association of serum renalase levels with indicators of glycolipid metabolism, blood pressure and sleep-related indicators.

| **Traits** | **β** | **p** | **β^*^** | **p^*^** |
| --- | --- | --- | --- | --- |
| AHI | 0.27 | **0.002** | 0.242 | **0.005** |
| ApoB | 0.244 | **0.007** | 0.223 | **0.013** |
| MAI | 0.203 | **0.023** | 0.189 | **0.035** |
| ODI | 0.265 | **0.003** | 0.232 | **0.007** |
| SBP |  |  |  |  |
| SBP (non-OSA) | 0.519 | **<0.001** | 0.488 | **<0.001** |
| SBP (severe OSA) | -0.297 | **0.019** | -0.307 | **0.026** |
| SBP (Total) | -0.01 | 0.914 | -0.043 | 0.616 |
| DBP |  |  |  |  |
| DBP (non-OSA) | 0.392 | **0.001** | 0.398 | **0.002** |
| DBP (severe OSA) | -0.337 | **0.007** | -0.345 | **0.012** |
| DBP (Total) | -0.043 | 0.629 | -0.079 | 0.365 |
| FPG | 0.069 | 0.441 | 0.033 | 0.709 |
| TC | 0.112 | 0.213 | 0.111 | 0.213 |
| TG | 0.007 | 0.939 | -0.024 | 0.790 |
| HDL | 0.015 | 0.866 | 0.057 | 0.507 |
| LDL | 0.07 | 0.436 | 0.071 | 0.422 |
| Insulin | 0.1 | 0.275 | 0.038 | 0.607 |
| ApoA | -0.129 | 0.158 | -0.108 | 0.238 |
| ApoE | 0.012 | 0.9 | -0.006 | 0.948 |
| HOMA | 0.071 | 0.442 | 0.007 | 0.930 |
| NC | 0.046 | 0.668 | 0.004 | 0.965 |
| WC | 0.074 | 0.493 | 0.02 | 0.768 |
| HC | -0.083 | 0.438 | -0.112 | 0.150 |
| WHR | 0.199 | 0.061 | 0.15 | 0.116 |
| ESS | 0.093 | 0.393 | 0.059 | 0.562 |
| Minimum SaO2 | -0.2 | 0.08 | -0.172 | 0.121 |

**Table S2.** Association of rs296545 with indicators of glycolipid metabolism, blood pressure and sleep-related indicators.

| **Traits** | **Non** | | | | **Mild** | | | | **Moderate** | | | | **Severe** | | | | **Total** | | | |
| --- | --- | --- | --- | --- | --- | --- | --- | --- | --- | --- | --- | --- | --- | --- | --- | --- | --- | --- | --- | --- |
|  | **β** | **p** | **β^*^** | **p^*^** | **β** | **p** | **β^*^** | **p^*^** | **β** | **p** | **β^*^** | **p^*^** | **β** | **p** | **β^*^** | **p^*^** | **β** | **p** | **β^*^** | **p^*^** |
| AHI | -0.031 | 0.442 | -0.017 | 0.661 | -0.042 | 0.486 | -0.044 | 0.464 | -0.043 | 0.209 | -0.04 | 0.247 | 0.013 | 0.511 | 0.017 | 0.368 | -0.009 | 0.568 | -0.018 | 0.358 |
| MAI | 0.002 | 0.95 | 0.007 | 0.859 | 0.09 | 0.134 | 0.094 | 0.12 | -0.008 | 0.824 | -0.006 | 0.866 | -0.015 | 0.445 | -0.015 | 0.455 | -0.012 | 0.444 | -0.016 | 0.345 |
| miniSO2 | 0.006 | 0.871 | 0.007 | 0.86 | -0.082 | 0.17 | -0.069 | 0.244 | 0.002 | 0.959 | 0.005 | 0.876 | -0.016 | 0.423 | -0.017 | 0.393 | -0.002 | 0.899 | 0.005 | 0.91 |
| averageSO2 | 0.056 | 0.162 | 0.052 | 0.193 | 0.025 | 0.678 | 0.035 | 0.558 | 0.051 | 0.138 | 0.07 | **0.038** | -0.018 | 0.358 | -0.018 | 0.344 | 0.01 | 0.527 | 0.016 | 0.341 |
| ODI | -0.007 | 0.853 | -0.008 | 0.837 | -0.004 | 0.951 | -0.017 | 0.771 | -0.045 | 0.192 | -0.046 | 0.183 | -0.018 | 0.366 | -0.015 | 0.424 | -0.022 | 0.152 | -0.03 | 0.064 |
| CT90 | -0.013 | 0.753 | -0.009 | 0.822 | 0.009 | 0.878 | 0.005 | 0.928 | -0.046 | 0.179 | -0.055 | 0.111 | 0.037 | 0.064 | 0.038 | 0.047 | 0.011 | 0.457 | 0.006 | 0.63 |
| SBP | 0.045 | 0.265 | 0.054 | 0.173 | -0.009 | 0.881 | -0.02 | 0.741 | -0.008 | 0.814 | -0.019 | 0.579 | 0.016 | 0.418 | 0.011 | 0.581 | 0.011 | 0.477 | 0.004 | 0.596 |
| DBP | 0.016 | 0.685 | 0.028 | 0.474 | -0.104 | 0.083 | -0.122 | **0.038** | -0.002 | 0.965 | 0.001 | 0.982 | 0.03 | 0.139 | 0.028 | 0.148 | 0.011 | 0.465 | 0.007 | 0.583 |
| FPG | -0.036 | 0.372 | -0.017 | 0.658 | -0.001 | 0.983 | -0.01 | 0.864 | 0.066 | 0.055 | 0.048 | 0.155 | 0.009 | 0.67 | 0 | 0.992 | 0.013 | 0.386 | 0.003 | 0.472 |
| TC | 0.011 | 0.778 | 0.022 | 0.576 | 0.082 | 0.172 | 0.074 | 0.218 | 0.036 | 0.289 | 0.045 | 0.189 | 0.004 | 0.843 | 0.007 | 0.72 | 0.014 | 0.353 | 0.013 | 0.398 |
| TG | -0.028 | 0.484 | -0.015 | 0.705 | 0.126 | **0.035** | 0.112 | 0.057 | 0.09 | **0.009** | 0.098 | **0.004** | 0.015 | 0.463 | 0.018 | 0.369 | 0.027 | 0.074 | 0.027 | 0.089 |
| HDL | 0.028 | 0.476 | 0.014 | 0.724 | -0.025 | 0.675 | -0.004 | 0.939 | 0.01 | 0.776 | 0.006 | 0.86 | -0.006 | 0.764 | -0.009 | 0.659 | 0.002 | 0.876 | 0.002 | 0.78 |
| LDL | 0.023 | 0.566 | 0.031 | 0.436 | 0.019 | 0.752 | 0.008 | 0.891 | -0.003 | 0.921 | 0.004 | 0.915 | -0.008 | 0.698 | -0.005 | 0.785 | -0.003 | 0.868 | -0.003 | 0.796 |
| APOA | -0.033 | 0.414 | -0.04 | 0.312 | 0.006 | 0.914 | 0.019 | 0.753 | 0.005 | 0.888 | -0.002 | 0.952 | -0.015 | 0.444 | -0.018 | 0.358 | -0.013 | 0.408 | -0.016 | 0.436 |
| APOB | 0.029 | 0.47 | 0.04 | 0.314 | -0.002 | 0.973 | -0.017 | 0.769 | 0.018 | 0.608 | 0.024 | 0.483 | 0.011 | 0.591 | 0.013 | 0.518 | 0.012 | 0.437 | 0.01 | 0.503 |
| APOE | -0.022 | 0.586 | -0.008 | 0.833 | 0.035 | 0.562 | 0.024 | 0.684 | 0.06 | 0.078 | 0.071 | **0.038** | 0.026 | 0.196 | 0.03 | 0.128 | 0.025 | 0.104 | 0.025 | 0.127 |

**Table S3.** Association of rs2296545 polymorphism with the hypertension risk in dominant and additive genetic models.

|  | Non OSA(n=629) | | Mild OSA(n=280) | | Moderate OSA(n=857) | | Severe OSA(n=2509) | |
| --- | --- | --- | --- | --- | --- | --- | --- | --- |
|  | OR (95%CI) | p | OR (95%CI) | p | OR (95%CI) | p | OR (95%CI) | p |
| rs2296545 |  |  |  |  |  |  |  |  |
| Dominant (CG+CC/GG) | 0.719(0.449-1.168) | 0.176 | 1.583(0.703-3.959) | 0.292 | 0.89(0.622-1.273) | 0.522 | 1.139(0.91-1.423) | 0.253 |
| Additive (CC/GG) | 0.724(0.423-1.242) | 0.238 | 1.871(0.77-4.977) | 0.184 | 0.778(0.52-1.165) | 0.222 | 1.269(0.992-1.623) | 0.057 |
| Additive (CG/GG) | 0.728(0.436-1.223) | 0.225 | 1.696(0.675-4.721) | 0.283 | 0.934(0.638-1.367) | 0.724 | 1.058(0.832-1.345) | 0.646 |
| Additive (CC/CG) | 0.991(0.645-1.517) | 0.967 | 1.439(0.788-2.639) | 0.237 | 0.838(0.608-1.153) | 0.278 | 1.258(1.047-1.512) | 0.014 |

Adjusted for age, BMI, FPG, TC, TG, HDL, LDL and LPa.

**Table S4.** Binding energy of renalase (37Glu) and renalase (37Asp) in combination with several catecholamines.

| Target | Catecholamines | Binding Energy（kcal/mol) |
| --- | --- | --- |
| Renalase（37Glu） | Adrenaline | -5.5054 |
| Renalase（37Asp） | Adrenaline | -5.0347 |
| Renalase（37Glu） | Norepinephrine | -5.2662 |
| Renalase（37Asp） | Norepinephrine | -5.0220 |
| Renalase（37Glu） | Isoprenaline | -6.4604 |
| Renalase（37Asp） | Isoprenaline | -5.6194 |
| Renalase（37Glu） | Dopamine | -5.2179 |
| Renalase（37Asp） | Dopamine | -4.8600 |
| Renalase（37Glu） | Dobutamine | -6.7809 |
| Renalase（37Asp） | Dobutamine | -6.7701 |

**Figure S1.** Rs2296545 makes the mutation of amino acid 37 from Glu to Asp, resulting in a smaller active pocket volume.


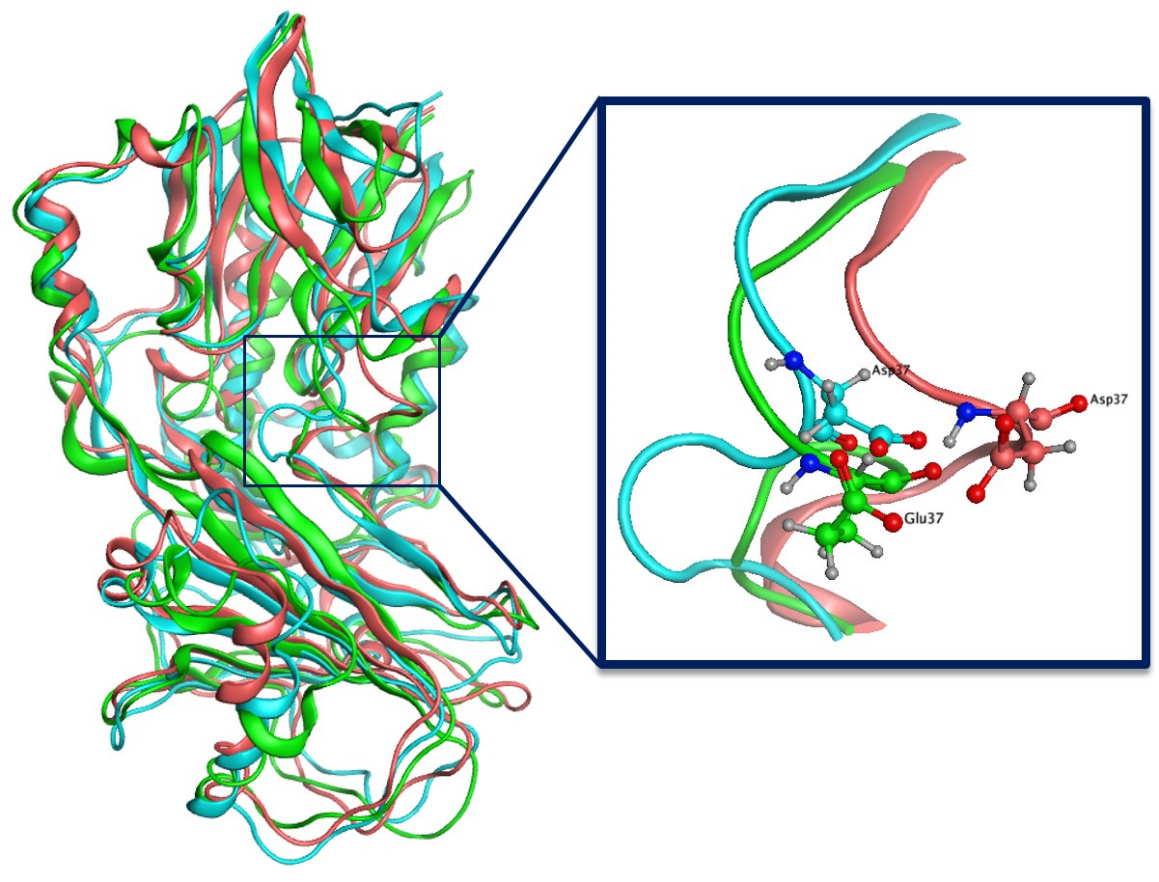

Supplement: Supplementary file 1 — Supplementary appendix [file 41440_2024_1850_MOESM1_ESM.docx]
